# Interaction of Liposomes Containing the Carrageenan/Echinochrome Complex with Human HaCaT Keratinocytes In Vitro

**DOI:** 10.3390/md22120561

**Published:** 2024-12-16

**Authors:** Ekaterina S. Menchinskaya, Vladimir I. Gorbach, Evgeny A. Pislyagin, Tatiana Y. Gorpenchenko, Evgeniya A. Pimenova, Irina V. Guzhova, Dmitry L. Aminin, Irina M. Yermak

**Affiliations:** 1 G.B. Elyakov Pacific Institute of Bioorganic Chemistry, Far-Eastern Branch of the Russian Academy of Sciences, 690022 Vladivostok, Russia; vigorbach@bk.ru (V.I.G.); pislyagin@hotmail.com (E.A.P.); daminin@piboc.dvo.ru (D.L.A.); 2 Federal Scientific Center of the East Asia Terrestrial Biodiversity, Far Eastern Branch of the Russian Academy of Sciences, 690022 Vladivostok, Russia; gorpenchenko@biosoil.ru; 3 A.V. Zhirmunsky National Scientific Center of Marine Biology, Far Eastern Branch, Russian Academy of Sciences, 690022 Vladivostok, Russia; eapimenova@yandex.ru; 4 Institute of Cytology of the Russian Academy of Sciences, 194064 Saint Petersburg, Russia; irina.guzh@gmail.com; 5 Department of Biomedical Science and Environmental Biology, Kaohsiung Medical University, Kaohsiung City 80708, Taiwan

**Keywords:** liposomes, carrageenan, echinochrome, microscopy, keratinocytes, *E. coli*, LPS, anti-inflammatory

## Abstract

Liposomal drug delivery systems are successfully used in various fields of medicine for external and systemic applications. Marine organisms contain biologically active substances that have a unique structure and exhibit a wide range of biological activities. Polysaccharide of red seaweed (carrageenan (CRG)), and water-insoluble sea urchin pigment (echinochrome (Ech)) interact with each other and form a stable complex. We included the CRG/Ech complex in liposomes for better permeability into cells. In our research, tetramethylrhodamine isothiocyanate TRITC-labeled CRG was synthesized to study the interaction of the complex encapsulated in liposomes with human epidermal keratinocytes (HaCaTs) widely used to expose the skin to a variety of substances. Using confocal microscopy, we found that liposomes were able to penetrate HaCaT cells with maximum efficiency within 24 h, and pre-incubation of keratinocytes with liposomes resulted in the delivery of the CRG/Ech complex into the cytoplasm. We investigated the anti-inflammatory effects of liposomes, including the lysosomal regulation, increased intracellular ROS levels, and increased NO synthesis in lipopolysaccharide (LPS)- or *Escherichia coli* (*E. coli*)-induced inflamed skin cells. Liposomes containing the CRG/Ech complex significantly reduced lysosomal activity by 26% in LPS-treated keratinocytes and decreased ROS levels in cells by 23% after LPS exposure. It was found that liposomes with the complex improved the migration of HaCaT keratinocytes incubated with high-dose LPS by 47%. The results of the work, taking into account the good permeability of liposomes into keratinocytes, as well as the anti-inflammatory effect on cells treated with LPS or *E. coli*, show the prospects of using liposomes containing the CRG/Ech complex as an anti-inflammatory agent in the fight against skin infections.

## 1. Introduction

Liposomal systems are widely used to increase the effectiveness and selectivity of drugs and reduce their toxicity in the treatment of various diseases and are considered a powerful drug delivery system due to their structural versatility as well as their biocompatibility, biodegradability, and non-toxic and non-immunogenic nature [1]. Liposomes as a drug delivery system have improved therapies for a range of biomedical applications by stabilizing therapeutic compounds, overcoming obstacles to cellular and tissue uptake, and improving the bio-distribution of compounds to target sites in vivo [2].

Liposomes have a significant impact as antibiotic carriers, improving the distribution of drugs and reducing their toxic properties [3,4], and are used to deliver anticancer drugs such as doxorubicin and vincristine [5]. Prescribing liposomes as drug delivery systems for treating infectious diseases and tumors has been well documented [5,6]. Liposomes, because of their ability to accumulate in the mononuclear phagocyte system, increase the bactericidal activity of drugs against intracellular pathogens [7]. Lipid vesicles are used to encapsulate active ingredients, providing better solubility, transport, and sustained release of the drug [8]. Liposomes, which consist of phospholipid bilayers, are microscopic vesicles ranging in size from 30 nm to several microns. The amphiphilic character of phospholipids in solution mimics natural cell membranes, allowing excellent interactions between liposomes and mammalian cell membranes promoting efficient cellular uptake [9]. A group of authors convincingly demonstrated that lipid vehicles are capable of transporting everolimus into cells, exerting the same molecular effect of the drug not only in LFs, but also in immune cells [10]. The drug loaded into the liposome is protected against physiologically occurring events, such as enzymatic degradation and chemical and immunologic inactivation, contributing to the improvement and extension of its action [11].

Marine organisms contain biologically active substances that are unique in structure and properties. Among marine organisms, algae have attracted special interest as a good source of bioactive substances, such as sulfated polysaccharides. These polysaccharides are extensively used in the medical and pharmaceutical fields due to their biocompatibility and biodegradability, mechanical stability, as well as scope for different chemical modifications [12].

Carrageenans (CRGs) are a group of sulfated galactans found in red seaweeds. They are highly valued for their structural diversity, which is associated with a wide range of physical, chemical, and biological properties [13]. The primary backbone structure of CRGs consists of alternating 3-linked β-D-galactopyranose and 4-linked α-D-galactopyranose residues. Different types of CRGs can be identified based on the structure of the repeating disaccharide units, the sulfation pattern, and the presence of 3,6-anhydrogalactose as a 4-linked residue [14]. The most commonly exploited types of CRGs in industrial applications are kappa (κ-), iota (ι-), and lambda (λ-) CRGs. These types differ in the number of ester-sulfate groups per repeating disaccharide unit, with kappa having one, iota having two, and lambda having three ester-sulfate groups. However, natural CRGs often contain a combination of these units [15]. The wide range of potential pharmacological applications of CRGs of different types has generated significant interest in these polysaccharides.

In recent years, the biological properties of CRGs such as their antiviral, anticoagulant, antioxidant, antibacterial, and antifungal action have been broadly studied [13]. These polysaccharides can interact and influence the immune response and therefore play a vital role in the treatment of many human diseases [16,17]. Antibacterial and antifungal effects of kappa-CRG isolated from alga *Hypnea musciformis* against *Staphylococcus aureus* and *Candida albicans* were found [18]. CRG has been shown to be a promising agent for reducing the transmission of ocular *Chlamydia* infection [19]. Complex therapy for patients with enteric infections caused by *Salmonella* has demonstrated the inhibitory effects of CRGs on LPS-induced inflammation [20]. CRGs have the ability to act as bioactive agents on their own, or enhance their bioactive capacity by binding to drugs or undergoing transformations or modifications to their chemical structure [16]. CRGs are ideal for various drug delivery applications. Previously, we used CRGs as a mucoadhesive matrix to include water-insoluble sea urchin pigment echinochrome A (7-ethyl-2,3,5,6,8-pentahydroxy-1,4-naphthoquinone (Ech)) [21]. The spectrum of biological protective effects of Ech is very diverse. The diverse range of protective effects of Ech in biology is primarily attributed to its capacity to counteract the adverse impact of free radicals [22]. The presence of Ech in combinations with CRG reduced its oxidation and increased solubility [21]. By computer simulation, we have shown that Ech interacts with CRG and forms a complex with it. We have demonstrated the anti-inflammatory effect of CRG/Ech complex in vivo for LPS-induced endotoxemia in mice and in vitro in LPS-stimulated RAW 264.7 cells and peritoneal macrophages. The CRG/Ech complex suppressed the LPS-induced inflammatory response, manifested by a decrease in ROS and NO production in macrophages and a decrease in the levels of pro-inflammatory cytokines in RAW 264.7 cells [23]. To possibly improve the effectiveness of the complex and obtain a form that can be easily applied, we incorporated the CRG/Ech complex into biodegradable liposomes.

We have previously characterized the physicochemical properties of liposomes containing the CRG/Ech complex by their Dynamic Light Scattering (DLS) and electrophoretic properties. We showed that Ech was not oxidized and retained stable after inclusion in liposomes and their lyophilization [24].

The incorporation of the CRG/Ech complex into liposomes and evaluation of their anti-HSV-1 effect has been shown by us recently. The liposome containing the CRG/Ech complex effectively reduced HSV-1 plaque formation after virus adsorption and penetration to cells. The virus-inhibiting activity of the liposomal form of the CRG/Ech complex was three times higher than that of the CRG/Ech complex itself [25].

Studying the interaction of various drug delivery systems and their structural components with cells is an important step in the development of new medical drugs. The purpose of this work was to study the interaction of liposomes containing a CRG/Ech complex with epidermal keratinocytes and to evaluate the anti-inflammatory effect of liposomes. To achieve this goal, fluorescently labeled CRG was synthesized and incorporated into liposomes. Incorporation of the CRG/Ech complex into liposomes may be useful for convenient oral administration of Ech with possible enhancement of its pharmacological action due to interaction with CRG.

## 2. Results

### 2.1. Characteristics of CRG, Ech, and Complex

CRG was extracted from the red seaweed *Chondrus armatus,* family Gigartinaceae, purified from low molecular weight impurities, and precipitated from solution by ethanol. Chemical analysis showed that the isolated polysaccharide contained galactose (Gal-40%), 3,6-anhydrogalactose (3,6 AnGal 16.2%), and a sulfate group (SO_4_^−2^ 27%) and had a molecular weight of 360 kDa determined by the viscometric method. According to spectroscopy data, this CRG consisted of kappa- and lambda-type CRGs in a ratio of 60:40, as we showed earlier [25]. The complex of Ech with CRG was formed as described in the Section 4. The ratio of the initial components CRG and Ech in the complex was 5:1 (*v*/*v*). Thus, 1 mL solution of the CRG/Ech complex contained 5 mg CRG and 1 mg Ech.

### 2.2. Preparation and Characteristics of Liposomes Containing CRG/Ech

**Figure 1:**
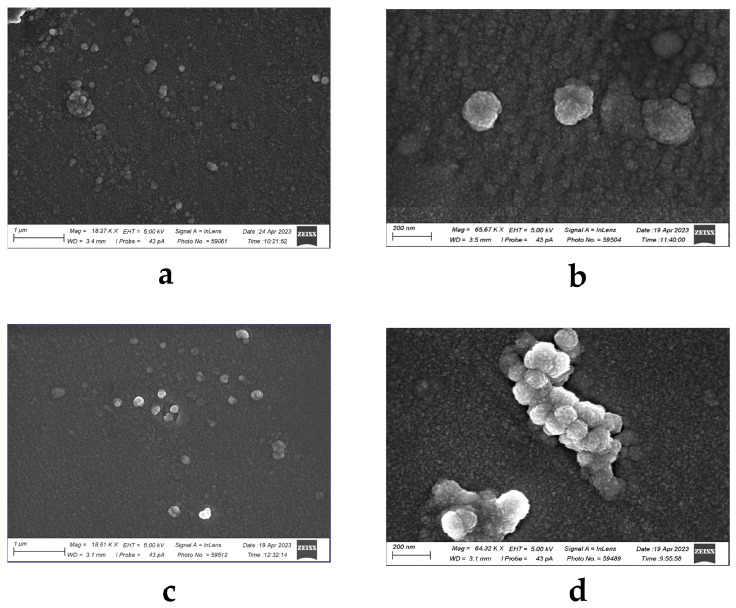
Scanning electron microscopy images of (**a**,**b**) conventional liposomes, (**c**,**d**) liposomes containing the CRG/Ech complex. Scale bars: 1 µm (**a**,**c**); 200 nm (**b**,**d**), EHT = 5.00 kV.

The applications of liposomes as drug delivery systems for the treatment of infectious diseases and tumors have been well documented [6]. Control (or empty) liposomes were produced using standard thin film hydration and the sonication method. Water solutions of the CRG/Ech complex were encapsulated into the liposome formulation using a standard thin film method followed by sonication. SEM micrography of the resulting liposomes is shown in Figure 1. As can be seen in the SEM images, all liposomes were spherical vesicles with a slightly rough surface, but different in size. Thus, the average diameter of control liposomes was 200 nm (Figure 1a); at the same time, a population of more homogeneous vesicles with a diameter from 100 to 150 nm was observed for CRG/Ech-containing liposomes (Figure 1b).

### 2.3. TRITC-Labeled CRG Containing Liposomes

TRITC-labeled CRG (CRG-TRITC) was synthesized to study the interaction of the CRG/Ech complex encapsulated in liposomes with cells. The number of amino groups in the molecule was determined by its reaction with picryl sulfonic acid according to the method of [26]. The content of amino groups was 42.1 residues per polymer molecule. Since the synthesis of CRG during the reaction with CCl takes place under severe alkaline conditions, partial desulfation of the polymer is possible. In this regard, the content of the sulfate group in the original and synthesized CRG was determined. According to the results obtained, the sulfate content in the activated sample was 97.15% of the original polymer, which indicates the unchanged CRG structure. Water solutions of CRG-TRITC were loaded into the liposome formulation using a standard thin film method followed by sonication.

### 2.4. Liposomes Penetrate and Accumulate in HaCaT Cells

**Figure 2:**
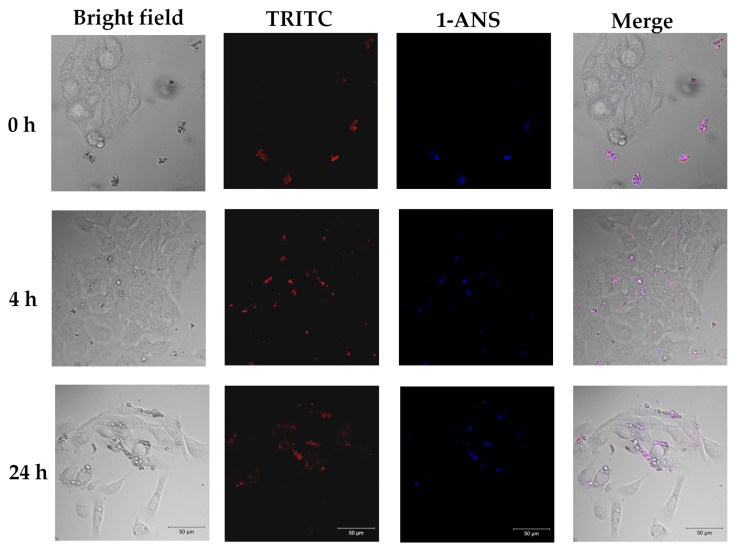
Localization of liposomes containing CRG-TRITC during incubation with HaCaT cells for 0 h, 4 h, and 24 h (appearance of HaCaT cells (B/W), blue color, 1-ANS (emission: 440–510); red color, TRITC (emission: 566–692); combined image; ×40). Scale bar = 50 μM.

Fluorescently labeled CRG-TRITC (100 μg/mL) was embedded inside liposomes (see the Section 4). The uptake of liposomes containing fluorescent CRG (CRG-TRITC) by HaCaT cells was assessed using a laser scanning confocal microscope. To more accurately localize liposomes in solution and analyze their delivery into cells, liposome membranes were stained with the fluorescent dye 1-ANS. The dynamics of liposome accumulation and their localization in cells were studied after 4 and 24 h of joint incubation (Figure 2). After 4 h of cultivation, we observed labeled liposomes in the culture medium and on the cell surface (Figure 2, 4 h). Over time, a redistribution of fluorescent signals was observed. An increase in the red signal was observed inside the cells (Figure 2, 24 h).

**Figure 3:**
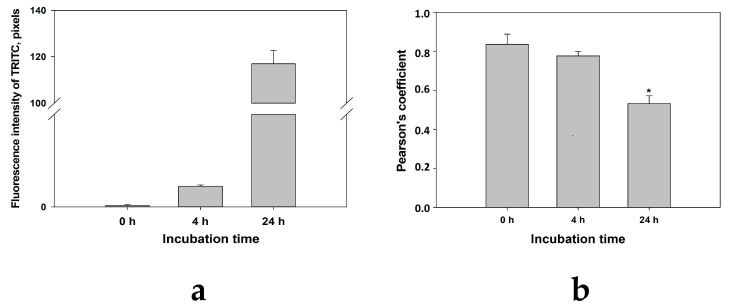
The fluorescence intensity value of liposomes containing CRG-TRITC after incubation with HaCaT cells (**a**). Colocalization analysis using the Pearson coefficient (between 1-ANS and TRITC) (**b**). Data are presented as mean ± SEM (*n* = 3). * *p* indicates a significant difference with *p* ≤ 0.05 compared to cells incubated with liposomes for 0 h.

To obtain more accurate information on the localization and accumulation of CRG in liposomes and inside cells, quantitative colocalization analysis was carried out using the coloc2 (Pearson’s correlation coefficient) package of the Fiji program (ImageJ), which provides all the important intensity correlation parameters. Pearson’s correlation coefficient is one of the colocalization coefficients for expressing the intensity correlation of pixel-wise colocalizing objects in each component of a dual-color image, often used in the analysis of confocal microscopy data. This coefficient indicates the degree of colocalization or the probability of the joint location of two different objects in one studied space. A decrease in the Pearson’s correlation coefficient value means the divergence and distance of two objects from each other. High correlation values of two spectrally different markers 1-ANS and TRITC in the same compartments (liposomes) at the starting point of the experiment (0 h) indicate the presence of CRG inside liposomes (Figure 3a). With the passage of incubation time, the value of the Pearson coefficient significantly decreased (Figure 3b). This is most likely due to the fact that the liposomes fuse with the cell membrane releasing TRITC-labeled CRG into the cell (Figure 3a). This is also confirmed by the increase in TRITC red fluorescence signal inside the cells with increasing incubation time (Figure 2, 24 h; Figure 3b).

**Figure 4:**
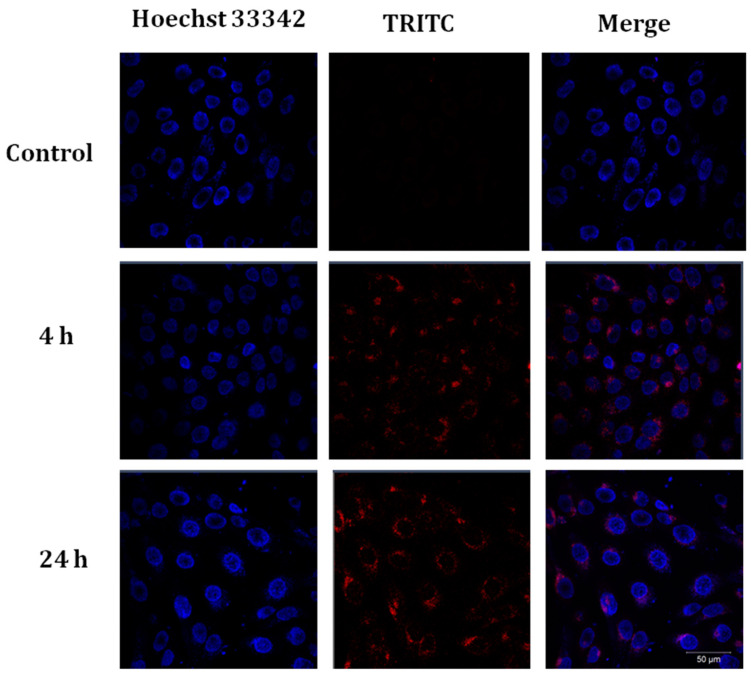
Fluorescent image of human HaCaT keratinocytes after incubation with liposomes containing the CRG-TRITC/Ech complex (100 μg/mL) for 4 and 24 h. Images were obtained by superimposing fluorescent channels. The fluorescence of liposomes with CRG/Ech is shown in red (TRITC), and cell nuclei are shown in blue (Hoechst 33342). Scale bar = 50 μM.

In the next step, liposomes containing the CRG-TRITC/Ech complex were fabricated. Registration of the fluorescence of cells with Hoechst 33342-stained nuclei revealed the appearance of the CRG-TRITC/Ech complex in the perinuclear region of cells after 4 h (Figure 4, 4 h). Over time, the intensity of the TRITC label was increased (Figure 4, 24 h). Thus, the maximal intensity of TRITC fluorescence in the cytoplasm was observed when cells were incubated with liposomes containing the CRG-TRITC/Ech complex for 24 h.

### 2.5. Cytotoxic Effect

**Figure 5:**
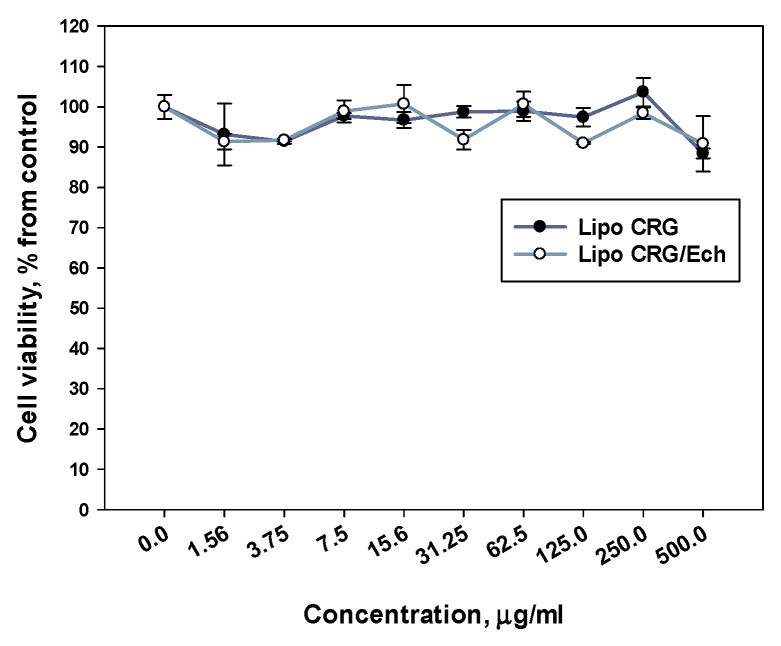
Effect of liposomes with CRG or CRG/Ech on HaCaT cell viability as measured by the MTT assay. HaCaT cells were treated with liposomes with CRG or CRG/Ech at concentrations of 1.56–500 μg/mL for 24 h.

Experiments assessing the cytotoxic activity of liposomes loaded with the CRG/Ech complex or only CRG against HaCaT cells showed that these liposomes did not suppress the viability of keratinocytes in the studied concentration range up to 500 μg/mL for 24 h of incubation (Figure 5).

### 2.6. Effect of Liposomes on Lysosomal Activity in HaCaT Cells

**Figure 6:**
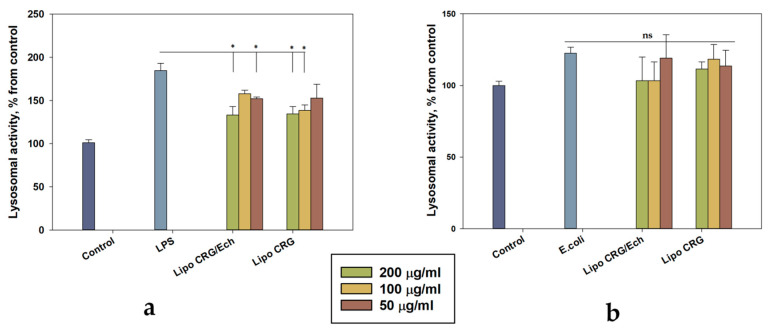
Lysosomal activity of liposomes containing the CRG/Ech complex or CRG pre-incubated with human keratinocytes (HaCaT cells) for 1 h. Cells (1 × 10^4^ cells/well) were incubated with LPS at 1.0 μg/mL (**a**) or *E. coli* 1 × 10^2^ CFU (**b**) for 24 h. The data are presented as the mean ± SEM values (*n* = 3); * *p* ≤ 0.05 indicates significant differences compared to cells exposed to LPS or *E. coli* alone; ns indicates that differences are not significant.

In a previous experiment, liposomes were shown to penetrate HaCaT keratinocytes and did not have cytotoxic activity. Then, HaCaT cells were pre-incubated with various doses of liposomes with the CRG/Ech complex or with CRG alone for 1 h before adding LPS (1 μg/mL) or *E. coli* (1 × 10^2^ CFU) for 24 h. Acridine orange (AO) staining is a well-established method for monitoring the stability of lysosomal membranes and lysosomal activity, using fluorescence microscopy [27], flow cytometry [28], or spectrofluorimetry [29]. As shown in Figure 6a, LPS causes an 84.5% increase in AO fluorescence in HaCaT cells compared to the control. Cells pre-incubated with liposomes containing the CRG/Ech complex at a concentration of 50 μg/mL and liposomes with CRG at a concentration of liposomes 100 and 200 μg/mL significantly reduced the effect of LPS on human keratinocytes by 17.5, 24.9%, and 26.2%, respectively. The greatest effect of 27.8% was observed for a concentration of 200 μg/mL for liposomes containing the CRG/Ech complex. The addition of *E. coli* (1 × 10^2^ CFU) to keratinocytes for 24 h increased lysosomal activity by 22.6% compared to the control. At the same time, liposomes with the CRG/Ech complex or with CRG showed a tendency to reduce AO fluorescence in cells (Figure 6b). The effect of empty liposomes on lysosomal activity in LPS-treated cells is shown in Figure S1.

### 2.7. Liposomes Suppress ROS and NO Production in LPS or E. coli-Treated HaCaT Cells

Furthermore, liposomes were assessed for their capacity to inhibit the cellular oxidative burst triggered by LPS or *E. coli*. To accomplish this, liposomes were co-cultured with cells an hour prior to the introduction of the inducer into the keratinocytes. Subsequently, the cells were incubated with LPS or *E. coli* for a period of 24 h. Following the incubation, the levels of reactive oxygen species (ROS) and nitric oxide (NO) production in the cells were measured using spectrofluorometric techniques.

**Figure 7:**
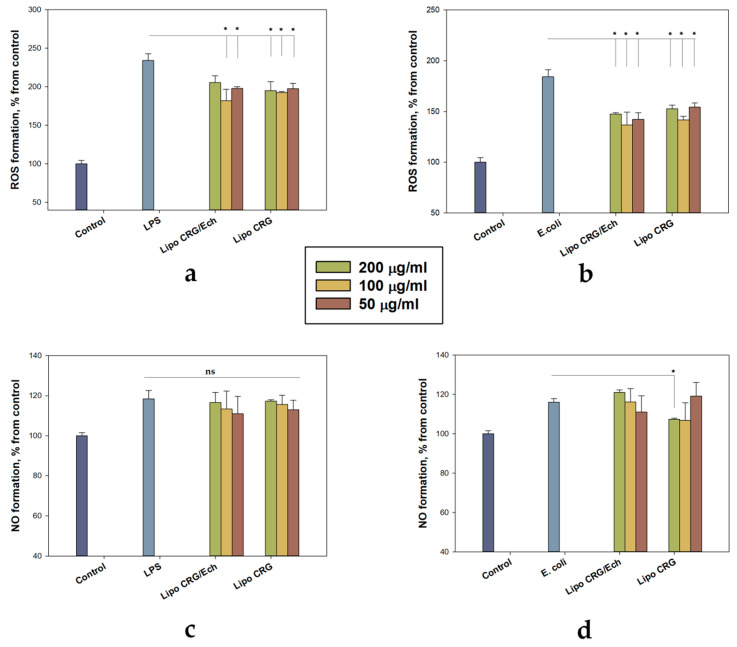
The effects of liposomes containing the CRG/Ech complex or CRG on the levels of ROS and NO production in HaCaT cells. Cells (1 × 10^4^ cells/well) were incubated with LPS at 1.0 μg/mL (**a**,**c**) or *E. coli* 1 × 10^2^ CFU (**b**,**d**) for 24 h. Intracellular ROS and NO levels were measured with H_2_DCF-DA and DAF-FM fluorescent probes, correspondingly, using spectrofluorimetry. Data are presented as mean ± SEM (*n* = 3). * *p* ≤ 0.05 indicates significant differences compared to cells exposed to LPS or *E. coli* alone; ns indicates that differences are not significant.

As shown in Figure 7, LPS and *E. coli* cause a marked increase in ROS and NO production in human keratinocytes. Thus, LPS increased the amount of ROS in keratinocytes by 134%. The application of *E. coli* to the cell culture of keratinocytes raised this indicator by 84%. It was found that liposomes significantly reduced ROS production in cells exposed to both LPS and *E. coli*. Liposomes with the CRG/Ech complex significantly eliminated the amount of ROS by 23% at all dosages tested, and liposomes with CRG lowered ROS production by 19% compared to control cells incubated with *E. coli*. Figure S2 shows a graph of the effect of empty liposomes (200 μg/mL) on the reduction of ROS levels in cells treated with LPS or *E. coli.* Pre-incubation of HaCaT keratinocytes with liposomes containing the CRG/Ech complex or CRG reduced NO production in LPS-exposed cells. The inhibitory effect was observed at all tested concentrations of liposomes. However, a statistically significant effect was shown for a concentration of 200 μg/mL liposomes with CRG when cells were incubated with *E. coli* (Figure 7d).

### 2.8. Liposomes Regulate Cell Migration in LPS-Treated HaCaT Cells

**Figure 8:**
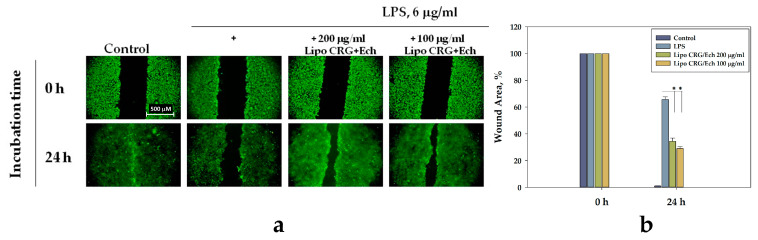
The effect of liposomes with the CRG/Ech complex on the migration of LPS-treated HaCaT cells (**a**). Migration of HaCaT cells into wound areas observed with a fluorescent microscope and processed by Image J 1.53t (**b**). Data are presented as means ± SEM (*n* = 3). * *p* ≤ 0.05 indicates significant differences compared to cells exposed to LPS alone. Scale bar = 500 μM.

It is known that high doses of LPS (up to 10 μg/mL) can inhibit the migration of cells, including keratinocytes [30]. We investigated the effect of liposomes containing the CRG/Ech complex on the migration of HaCaT cells incubated with LPS. For this purpose, special devices from Ibidi^®^ were used to create a cell-free zone in a monolayer of HaCaT cells. The cells were stained with the fluorescent dye CFDA-SE, after which liposomes containing the CRG/Ech complex were added at different concentrations, and after 1 h, LPS (6 μg/mL) was added to the cells. The migration of keratinocytes was observed by fluorescent microscopy for 24 h (Figure 8a).

A significant inhibition of keratinocyte migration due to the influence of high concentrations of LPS (6 μg/mL) was found, while complete fusion of the cell-free zone in untreated control cells was observed. All tested liposomes significantly improved the migration of cells incubated with LPS into the cell-free zone (Figure 8a). The CRG/Ech complex encapsulated in liposomes had a stimulatory effect on the cell-free fusion zone; this effect was significantly different from cells incubated with LPS. The increase in keratinocyte migration was 55.89% and 47.76% for liposomes containing the CRG/Ech complex at liposome concentrations of 100 and 200 μg/mL, respectively (Figure 8b).

## 3. Discussion

In recent years, liposomal systems have been used in medical practice for external and systemic applications. Such systems are successfully used due to their bioavailability and ease of preparation. In addition, they provide controlled release of the drug [11]. Natural polysaccharides, due to their biocompatibility, biodegradability, and mechanical stability, are widely used to create various biomedical systems and biomaterials [13,16]. Among them, the sulfated polysaccharide CRG attracts special attention due to its biological activity, favorable physicochemical properties, and the presence of functional groups [31,32].

Consequently, CRGs are ideal for various drug delivery applications that take advantage of their mucoadhesive properties [33]. Additionally, the high water adsorption ability of CRGs can enhance drug dissolution, thereby increasing the oral bioavailability of poorly water-soluble medications.

Earlier, we have shown that CRGs improve the water solubility of Ech and formers with its CRG/Ech complex [23,24,25]. Ech is the active substance (P N002362/01) of the drug histochrome, which is officially registered in the Russian Federation and is a successfully used drug with no identified side effects. However, histochrome is available only in the form of an injection solution in ampoules, which greatly limits the possibilities of its use. Most failures in the development of new drugs are usually due to the poor solubility of the active ingredient in water and its low stability. As we have previously shown, Ech in the complex with CRG does not oxidize and remains stable after incorporation into liposomes and after the lyophilization process [24].

In this work, we obtained liposomes containing the CRG/Ech complex and studied the interaction of these liposomes with human epidermal keratinocytes (HaCaTs).

The analysis of morphological characteristics, namely the shape, is vital for an adequate characterization of liposomes. The most selected tool to ascertain the morphological features of liposomes is microscopy [34]. It is known that the size of liposomes affects their pharmacokinetics, tissue extravasation, tissue diffusion, hepatic uptake, kidney excretion, and clearance rate from the site of injection. Only liposomes of a mean diameter between 100 and 150 nm are able to enter fenestrated vessels in the liver endothelium, secondary lymphoid structures, or tumor microenvironments [13]. The cell uptake is most relevant to liposomes with a diameter of 100–150 nm [35]. The visualization of liposomes as individual particles by microscopy techniques provides a direct observation of their shape. In our case, a population of homogeneous liposomes containing the CRG/Ech complex with sizes from 100 to 150 nm was observed. These liposomes at concentrations up to 500 μg/mL did not exhibit cytotoxicity towards keratinocytes.

The similarity of liposomes to the cell membrane and their ability to include the CRG/Ech complex with no change in its chemical nature make liposomes a convenient drug delivery system in cells. Obtaining TRITC-labeled CRG made it possible to evaluate the dynamics of the accumulation of liposomes containing the CRG/Ech complex out on human keratinocytes (HaCaTs). In this study, it was shown that the CRG/Ech complex or CRG alone encapsulated in liposomes was able to pass through and accumulate in human HaCaT keratinocytes. According to the data obtained, the best interaction of liposomes with cells was observed during their daily incubation. Analysis of the colocalization of fluorescent markers of liposome membranes (1-ANS) and CRG-TRITC indicates that after 24 h only the TRITC label is located inside the cells, while 1-ANS is localized on the surface of keratinocytes. This could mean that after contact with cells, liposomes fuse with the cell biomembranes, but the CRG/Ech complex penetrates into the cells and is concentrated in the cytoplasm.

Infections of the skin and soft tissues and the inflammation associated with this pathogenic process are common problems for people of all ages. These diseases are mainly associated with the impact on the skin of pathogens such as *Staphylococcus aureus* and streptococci, but it has been repeatedly mentioned that *E. coli* is also involved in the infection of dermis wounds. Natural products are one of the most popular sources of complementary and alternative medicines for preventing and treating inflammatory disorders. For example, Ech is known to have beneficial effects against various diseases. Through its antioxidant capacity, Ech is known to decrease LPS-induced ROS production in kidney epithelial cells [36]. It was demonstrated that Ech was a strong inhibitor of melanogenesis, and was effective in preventing pigmentation and promoting skin whitening. Ech protects the intestinal epithelium from oxidative damage and promotes its regeneration by regulating the expression of stem cell genes [37].

It is also known that sulfated algal polysaccharides suppress the inflammatory response induced by LPS, manifested by a decrease in NO and PGE2 production, inhibition of iNOS and COX-2 expression, and decreased levels of pro-inflammatory cytokines [38]. CRGs can be considered anti-endotoxin agents that neutralize LPS and eliminate their pro-apoptotic activity [39]. As we have earlier shown, CRG exhibits an anti-inflammatory effect in LPS-induced endotoxemia. The CRG/Ech complex also suppressed the LPS-induced inflammatory response displayed in reducing the production of ROS and NO in macrophages [23]. Taking into account the diversity of activities of Ech and CRG, as well as the possible synergism of their action, we obtained a new type of this complex in the form of liposomes. In this work, we obtained liposomes containing the CRG/Ech complex and tested their ability to inhibit the inflammatory effect caused by both LPS and the bacteria *E. coli* on human keratinocytes (HaCaT cells).

In the present study, the effect we discovered of the CRG/Ech complex encapsulated in liposomes on induced inflammation of keratinocytes is close to the effect of the known anti-inflammatory reference compounds aspirin and curcumin, which suppress the pro-inflammatory activity of LPS and TNF-α + INF-γ [40,41].

In addition, the purpose of the present study was to investigate the anti-inflammatory activities of the liposomes, including lysosomal regulation, intracellular ROS level elevation, and NO synthesis up-regulation in inflamed skin cells. LPS or *E. coli* were used as inflammatory agents in our study. We investigated the anti-inflammatory effect of liposomes containing the CRG/Ech complex in different models of cell disorders and compared it with the effect of liposomes containing only CRG. Pre-incubation of cells with liposomes led to a significant decrease in lysosomal activity. CRG/Ech encapsulated in liposomes at a low studied concentration of 50 μg/mL reduced the effect of LPS on keratinocytes by 17%, while a similar effect of CRG liposomes was observed at their concentration two times higher. Liposomes were able to enhance the migration of LPS-treated keratinocytes reflecting a wound healing effect.

ROS are the products of oxygen metabolism and play an important role in cell signaling and homeostasis. High levels of ROS may result in significant damage to the cell structure and lead to so-called oxidative stress [26]. In the present work, the ability of liposomes to suppress the oxidative burst in HaCaT cells induced by both LPS and *E. coli* cells was obtained. It was found that liposomes containing CRG/Ech complex statistically significantly reduced ROS production and the NO level. Liposomes containing only CRG were less effective in this experiment.

Migration and proliferation of HaCaT cells were suppressed by a high concentration of LPS, which is consistent with previous studies demonstrating the inhibitory effect of LPS [30,42]. When liposomes containing CRG/Ech complex were administered, migration and proliferation were restored, as the complex possesses antioxidant properties, reducing LPS-induced oxidative stress in keratinocytes. As a result, we observed a noticeable increase in the motility of keratinocytes and a rise in the speed of their movement into the wound area. This effect is certainly associated with an improvement in the physiological state of LPS-treated cells in the presence of liposomes containing the CRG/Ech complex.

The prevention of diseases caused by free radicals is greatly influenced by antioxidant substances. In this work, we used a complex of antioxidant Ech with CRG, since such a complex is water-soluble, unlike the individual echinochrome A. It was the acquisition of water-soluble properties that made it possible to easily enclose such a complex into liposomes without losing the biological activity of the Ech itself.

The high activity of liposomes containing the CRG/Ech complex to decrease LPS-induced inflammation in skin cells is primarily due to the antioxidant capacity of Ech and the anti-endotoxin effect of CRG. Pre-incubation of keratinocytes with liposomes containing the CRG/Ech complex leads to delivery of this complex to the cytoplasm, a decrease in cellular lysosomal activity, a reduction of the ROS and NO level, and enhances the migration of inflammatory agent-treated skin cells.

## 4. Materials and Methods

### 4.1. Extraction of Carrageenan (CRG)

The red algae *Chondrus armatus* (the vegetative form) were harvested in Peter the Great Bay, Sea of Japan. The alga (50 g) was treated with acetone to remove pigment and then suspended in hot water (1.5 L). The polysaccharide was extracted at 90 °C for 2 h in boiling water; this procedure was repeated three times. The residues were removed by centrifugation and the crude polysaccharides were purified by redissolving in water, filtered through a Vivaflow200 membrane (Sartorius, Gottingen, Germany) with a pore size of 100 kDa, concentrated, and poured into ethanol (three volumes). The lyophilized yielding polysaccharide (CRG) was 50% and the structure was established according to a published protocol [25].

Monosaccharides as alditol acetate derivatives were identified by gas chromatography (GC) using an Agilent 6850 gas chromatograph (Santa Clara, CA, USA) equipped with a HP-5MS capillary column (30 m × 0.25 nm) with 5% phenylmethylsiloxane and a flame ionization detector. The analyses were carried out at a temperature programming from 175 to 225 °C with 3° min^−1^ [43,44]. The content of 3,6-anhydrogalactose was determined according to the method of Usov and Elashvili [45] and sulfates by the turbidimetric method of Dodgson and Price [46]. Viscosimetric molecular weights of CRG was calculated using the Mark–Houwink equation: [η] = KMα, where [η] is the intrinsic viscosity and K and α are empirical constants constituting 3 × 10^−3^ and 0.95 at 25 °C in 0.1 M NaCl for carrageenans [47].

Commercial LPS was from the bacterium *Escherichia coli* 103 055:B5 (Catalog No. L2880, Lot No. 102M4017V, Sigma, St. Louis, MO, USA).

### 4.2. Labeling of Carrageenan with TRITC

CRG (48 mg) was dissolved in water (4.8 mL) by heating at 50 °C for 2 h and a solution of a mixture of 0.25 N NaOH with 0.5 M NaHCO3 pH 10.1 (4.8 mL) was added. Then a solution of 30 mg cyanuric chloride (CCl) in 1 mL dimethyl sulfoxide (DMSO) was added, stirred for 5 min, and 1 mL solution of 1,4-diaminobutane (DAB) in water (20 mg/mL) was added. The resulting mixture was left overnight at 20 °C. The solution was dialyzed through a MEMBRA-CEL MD25-14 × 1000 CLR membrane 5 times against distilled water, evaporated to 5 mL, and lyophilized. The yield of aminated polysaccharide was 47.9 mg.

A sample of the resulting aminated polysaccharide (45 mg) was dissolved in 5 mL of water, a solution of 3.1 mg of tetramethylrhodamine -5-(and -6)- isothiocyanate (5/6-TRITC mixed isomers) (T490, Sigma) in 5 mL of DMSO and then 50 μL of triethylamine was added. The mixture was kept for 4 h at 20 °C and dialyzed 5 times through a MEMBRA-CEL MD25-14 × 1000 CLR membrane against distilled water. The solution after dialysis was evaporated to 6 mL and passed through a 50 × 1.5 cm column with Sephadex G-25 sorbent (Fine, Pharmacia Fine Chemical AB, Uppsala, Sweden). Water was used as the eluent. Fractions were analyzed spectrophotometrically for the content of the TRITC group at D535 nm and fluorimetrically at Dex/Dem 530/590 nm. Fractions matching the maximum absorption and fluorescence were collected and lyophilized. The yield of the resulting TRITC polysaccharide was 39.9 mg (88.7%). The number of TRITC groups per labeled CRG molecule (12) was calculated based on the molar extinction coefficient—8.1 × 10^4^.

### 4.3. Preparation of CRG/Ech Complex

Echinochrome (Ech) was isolated from sea urchins *Scaphechinus mirabilis* as described in [48] and was used as a stock solution in ethanol at concentration 10 mg mL^−1^. Ech was added to 0.1% aqueous solutions of CRG to a concentration of 0.1 mg mL^−1^ and the mixture was stored in the dark at 37 °C. Thus, a solution of Ech in carrageenan with a component ratio of 1:5 (*w*/*w*) was obtained for work.

### 4.4. Preparation of Liposomes

The 0.24 mL solution of the L-α phosphatidyl choline (Egg chicken, 840051, Avanti Polar Lipids, Alabaster, AL, USA) (100 mg/mL) and the 0.15 mL solution of cholesterol (Sigma Grade ≥ 99%, c 8667) and the 0.15 mL solution of cholesterol (70 mg/mL) were prepared in chloroform-methanol 9:1 (*v*/*v*) and the mixture was evaporated to a film on the glass test tube and kept under vacuum to remove residual solvent. A thin film of lipids in a glass test tube was obtained by evaporating their chloroform–methanol solution on a BUCHI Heating Bath B-490 rotary evaporator and then drying the sample in a vacuum pump (0.2 Pa) for 2 h.

### 4.5. Conventional Liposomes

A mixture of dried lipid film and 1 mL of water was treated with ultrasound twice (15 min) in an ultrasonic apparatus (Elmasonic S, Elma GmbH & Co. KG, Singen, Germany); the resulting solution was centrifuged (15 min at 15,000× *g*) and the supernatant was removed and the sediment was suspended in 5 mL water as described previously [24]. Liposomes were lyophilized in suspended vials.

### 4.6. CRG- and CRG/Ech-Containing Liposomes

An amount of 1 mL water solution of 0.5% carrageenan (CRG-containing liposomes) or 1 mL solution of CRG/Ech complex (CRG/Ech-containing liposomes) was added to the dried lipid films. The mixtures were sonicated 2 times for 15 min in an ultrasonic batch (Elmasonic S, Elma GmbH & Co. KG, Singen, Germany) and then centrifuged for 15 min at 15,000× *g*. The supernatants were removed, the pellets were suspended in water, and centrifuged again as described above and in [24].

The stability of Ech in the liposomes was determined by measuring the absorption value at 468 nm. For hydration, vials with a sample of dry liposomes were suspended in water at a concentration of 2 mg/mL for 20 min in an ultrasonic bath.

### 4.7. Measurement of Encapsulation Efficiency

Water (0.4 mL) and n-butanol (0.4 mL) were added to the suspension of liposomes (0.1 mL).

The mixture was acidified with 20 µL of a 3 M HCl solution, shaken, incubated for 10 min in a US bath, and centrifuged for 20 min at 15,000× *g*. The upper butanol liquid layer was used to determine the Ech, and the aqueous layer was used to determine the content of carrageenan. The concentration of Ech in the solution was determined by spectrophotometry (Unicam 2 UV/VIS spectrophotometer) using absorption spectra at λ = 468 nm. The content of the encapsulated Ech (Y, mg) was estimated from the difference between the total used drug (X) and the released drug. Thus, the encapsulation efficiency (EE) was calculated according to the equation EE% = X/Y × 100%, and a calibrated straight line was constrained in the same condition.

The content of carrageenan-loaded liposomes was determined using a Taylor reagent (1,9-Dimethyl-methylene Blue, Sigma-Aldrich, Saint Louis, MO, USA). To this, 40 µL of aqueous extract was added per 200 µL of reagent. The mixture was incubated for 10 min, and the optical density was measured on a spectrophotometer (Bio-Tec Instruments, Inc., Auburn, CA, USA) at 535 nm.

### 4.8. Preparation of TRITC-CRG- and ANS-Containing Liposomes

The solutions of TRITC-CRG 1 mg/mL in water and 8-anilino-1-naphtalene-3-sulfonic acid (ANS 13.99020, Aldrich Chem. Co) 1 mg/mL in methanol were prepared. An amount of 0.05 mL of this solution was diluted with 0.95 mL of water to a 50 μg/mL concentration of ANS. The liposomes were obtained with mixture of 0.5 mL solution TRITC-CRG and 0.5 mL water, or with a mixture of 0.5 mL solution of ANS and 0.5 mL water, or a mixture of 0.5 mL solution TRITC-CRG and 0.5 mL solution of ANS as described above for CRG/Ech-containing liposomes. The concentration of the resulting hydrated liposomes was 1 mg/mL.

### 4.9. The Hydration of Liposomes

To obtain a suspension of liposomes, water or aqueous solutions of the studied components were added to the dried lipid film in the test tube and the test tube was sonicated in an ultrasonic generator at power 36 KHz for 20 min. Then, the liposome suspension was centrifuged at 15,000× *g*, the supernatant was discarded, and the sediment was suspended in water and centrifuged again. The procedure was repeated 3 times. The resulting liposome sediment was suspended in water and lyophilized in weighed vials, thus determining the weight of each sample portion. Hydration of the lyophilized samples was performed by adding a certain volume of water to the vial and sonicating it in an ultrasonic generator at power 36 KHz for 20 min.

To study liposomes, their suspension in water was obtained. For hydration, the samples of dry liposomes were sonicated in water an ultrasonic bath for 30 min at concentration of 1 mg/mL.

### 4.10. Scanning Electron Microscopy (SEM)

The liposomes present on cover slides were fixed using a solution of 4% (*v*/*v*) glutaraldehyde in 0.1 M phosphate buffer at pH 7.2 for a duration of 2 h at room temperature. The cells were then dehydrated by passing them through a series of ethanol and the ethanol was subsequently replaced with isoamyl acetate. Sections of the cover glass containing cells were dried using K850 critical point dryers from Quorum Technologies, London, UK. Mounted on stubs, coated with carbon, the dried sections underwent examination using a Zeiss Sigma 300 VP SEM from Carl Zeiss Ltd., located in Cambridge, UK.

### 4.11. HaCaT Cell Culture

HaCaT cells (human keratinocytes) obtained from the Cancer Research Center (Heidelberg, Germany, from Prof. N. Fusenig) were grown in DMEM containing 10% fetal bovine serum and 1% penicillin/streptomycin (Biolot, St. Petersburg, Russia). HaCaT cells were incubated at 37 °C and 5% CO_2_.

### 4.12. Fluorescence Confocal Microscopy

HaCaT keratinocyte cells were placed in a 6-well plate at a concentration of 5 × 10^4^ cells/mL and left for 24 h for adhesion. Before sowing, one glass cover glass measuring 30 × 0.17 mm (Paul Marienfeld GmbH & Co. KG, Lauda-Königshofen, Germany) was placed in each well. After this, the studied liposomes were added to the cells and further incubated for 0 h, 4 h, and 24 h. The accumulation of liposomes with CRG/TRITC as well as liposomes with CRG/Ech+TRITC in HaCaT cells was studied using a laser scanning confocal microscope LSM 710 LIVE Axio Observer (Carl Zeiss GmbH, Jena, Germany). Fluorescence of 1-ANS or Hoechst 33,342 was excited by a 405 nm laser, and emission was recorded in the range of 440–510 nm; TRITC detection was excited by a laser with a wavelength of 543 nm and recorded in the emission range 566–692 nm. Cell image processing and subsequent analysis were performed using ZEN 2011 software (Carl Zeiss GmbH, Jena, Germany) and the free image processing software Fiji (ImageJ, 1.53 t, Wayne Rasband and contributors, National Institutes of Health, Kensington, CA, USA). For the analysis of the colocalization, the JACoP plugin (Just Another Colocalization Plugin) and the Pearson’s coefficient were used. The samples were examined at the Center for Collective Use “Biotechnology and Genetic Engineering” of the Federal Scientific Center for Biodiversity, Far Eastern Branch of the Russian Academy of Sciences.

### 4.13. MTT Cytotoxicity Test

The toxic effects of liposomes with CRG/Ech complex or CRG on HaCaT cells were assessed using a MTT assay. To summarize, HaCaT cells were distributed into a 96-well plate (10 × 10^3^/per well) and incubated for 24 h for adhesion. Subsequently, the cells were treated with varying concentrations of liposome ranging from 0 to 500 μg/mL for an additional 24 h. Following this, the medium was replaced with fresh DMEM, and 10 μL of MTT solution (Sigma-Aldrich, St. Louis, MO, USA) (5 mg/mL) was introduced to each well, leading to a further 4 h incubation. The supernatant was then discarded, and the purple sediment in each well was solubilized with 100 μL of SDS-HCl solution over 18 h. The optical density of the resulting formazan dye was quantified using a Multiskan FC microplate photometer at a 570 nm wavelength. These procedures were performed three times.

### 4.14. Lysosomal Activity

Evaluation of lysosomal function within HaCaT keratinocytes was conducted by marking the lysosomes with acridine orange dye and subsequent analysis. The keratinocytes were placed into 96-well plates at a density of 10 × 10^3^ cells/well and were incubated at 37 °C in an atmosphere containing 5% CO_2_ for 24 h. Following cell attachment, the keratinocytes were exposed to various liposome concentrations for 1 h, after which they were treated with either 1 μg/mL of LPS or *E. coli* at 10^2^ CFU, and incubated for an additional 24 h. To assess lysosomal function, 20 μL of acridine orange solution (initial concentration 10 μg/mL) was added to each well and the cells were further incubated for another 30 min at 37 °C; then, the cells were washed three times with PBS and 200 μL of PBS was added to each well. Fluorescence measurements were performed at excitation/emission wavelengths of 485/590 nm on a PHERAstar FS plate reader (BMG Labtech, Ortenberg, Germany).

### 4.15. ROS and NO Level Analysis

The analysis of ROS and NO levels was conducted on HaCaT cells, which were seeded at a density of 1 × 10^4^ cells per well and allowed to adhere for 24 h. These cells were then treated with liposomes containing either the CRG/Ech complex or CRG alone at doses of 50, 100, and 200 µg/mL for one hour. Subsequently, LPS at a concentration of 1 µg/mL or *E. coli* at 10^2^ CFU were introduced to each well, followed by a further 24 h incubation period.

To assess the production of reactive oxygen species (ROS), a 20 µL aliquot of 2,7-dichlorodihydrofluorescein diacetate was added to each well to reach a concentration of 10.0 µM. The wells were then incubated for 10 min at 37 °C in the absence of light.

In the case of nitric oxide (NO) quantification, the wells received treatment with a DAF-FM fluorescent probe. Subsequently, the plate underwent a 40-min incubation at 37 °C in darkness. The fluorescence was quantified using a PHERAstar FS high-speed plate reader (BMG Labtech, Ortenberg, Germany), with measurements taken at an excitation wavelength of 485 nm and emission wavelength of 518 nm. The data were analyzed with MARS Data Analysis software version 3.01R2, and the findings were expressed as a percentage of the positive control.

### 4.16. Cell Migration

A laboratory-based wound repair test was employed to observe the movement of live HaCaT cells. To evaluate the impact of liposomes infused with CRG/Ech or CRG on the movement of keratinocytes (at a density of 5 × 10^5^ cells/mL) activated by LPS, special well inserts (Culture-insert 2 Well 24, ibidi GmbH, Martinsried, Germany) were utilized and then taken out, creating a separation of 500 ± 50 μm as specified by the producer, between the adhering cells (an area devoid of cells). Following the extraction of the inserts, the cells underwent a double rinse with PBS to clear away any cellular remnants and unattached cells, and were then stained with the fluorescent marker CFDA-SE (5,6)-carboxyfluorescein diacetate succinimidyl ester (LumiTrace CFDA-SE kit, Lumiprobe, Moscow, Russia). This was achieved by treating the cells with a 10 µM PBS solution for 5 min at a temperature of 37 °C; subsequently, the cells were cleansed twice more with PBS and replenished with new growth medium. Thereafter, the cells received treatment with varying doses of vesicles containing CRG/Ech or CRG; following a period of one hour, LPS (at a concentration of 6 μg/mL) was introduced and left to interact for 24 h. Cells that were only exposed to the growth medium served as the baseline control. The migration of cells into the wounded region was then monitored using a fluorescent microscope (MIB-2-FL, LOMO, Russia) equipped with a 20× lens.

### 4.17. Statistical Analysis

All experiments were performed in triplicate. Data were subjected to statistical analysis using one-way ANOVA tests. Data were shown as mean ± SEM, and *p* ≤ 0.05 was regarded as statistically significant. All statistical tests were performed using SigmaPlot 14.0 software (Systat Software Inc., San Jose, CA, USA).

## 5. Conclusions

The CRG/Ech complex or CRG encapsulated in liposomes is able to penetrate and accumulate in human keratinocytes. In addition, pre-incubation of cells with liposomes leads to a decrease in lysosomal activity and a decrease in ROS and NO in skin cells after the incubation with LPS or *E. coli*. They also significantly enhance the migration of keratinocytes depressed with high concentrations of LPS. Given the good permeability of liposomes into keratinocytes, as well as the anti-inflammatory effect on cells treated with LPS or *E. coli*, the use of such liposomes containing the CRG/Ech complex as an anti-inflammatory agent in the fight against skin infections deserves further study.

Furthermore, inflammatory diseases are usually concomitant with bacterial and viral infections. The complex, as we have shown, has both anti-inflammatory and antiherpetic effects and can be useful for bacterial and viral infections. Inclusion of this complex in liposomes will allow it to be used in a more convenient form for oral, buccal, or nasal administration.

## Data Availability

The original contributions presented in the study are included in the article, further inquiries can be directed to the corresponding authors.

## Supplementary Materials

The following supporting information can be downloaded at: https://www.mdpi.com/article/10.3390/md22120561/s1, Figure S1: Lysosomal activity of free liposomes (200 mg/mL) pre-incubated with human keratinocytes HaCaT cells for 1 h. Cells (1 × 104 cells/well) were incubated with LPS at 1.0 μg/mL or *E. coli* 1 × 102 CFU for 24 h; Figure S2: The effects of free liposomes (200 μg/mL) on the levels of ROS. Cells (1 × 104 cells/well) were incubated with LPS at 1.0 μg/mL or *E. coli* 1 × 102 CFU for 24 h; Table S1: Summary of the anti-inflammatory activity of liposomes containing CRG/ECh complex or CRG when keratinocytes were treated with LPS or *E. coli* for 24 h.
