# Supplementary material for: Interaction of Liposomes Containing the Carrageenan/Echinochrome Complex with Human HaCaT Keratinocytes In Vitro
Source: Mar Drugs. 2024 Dec 16;22(12):561. doi: 10.3390/md22120561 (PMC11676407; doi:10.3390/md22120561)
Supplement: Supplementary file 1 [file marinedrugs-22-00561-s001.zip › marinedrugs-3325753-supplementary.pdf]

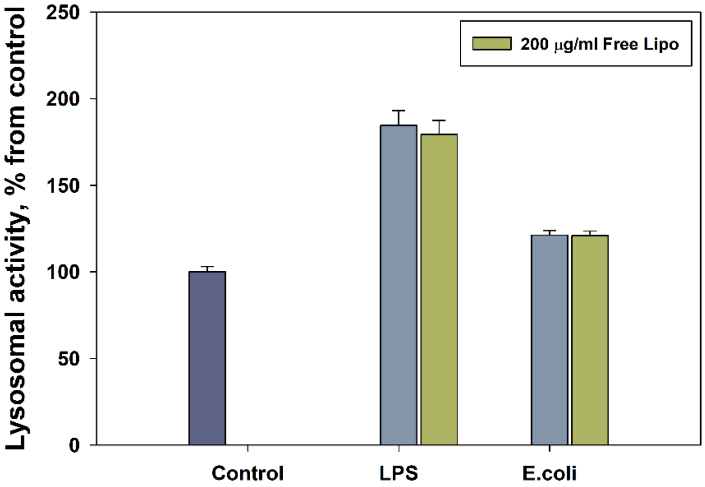

Figure S1. Lysosomal activity of free liposomes (200 mg/ml) pre-incubated with human keratinocytes HaCaT cells for 1 h. Cells (1 × 10^4 cells/well) were incubated with LPS at 1.0 μg/mL or *E. coli* 1 × 10^2 CFU for 24 h.

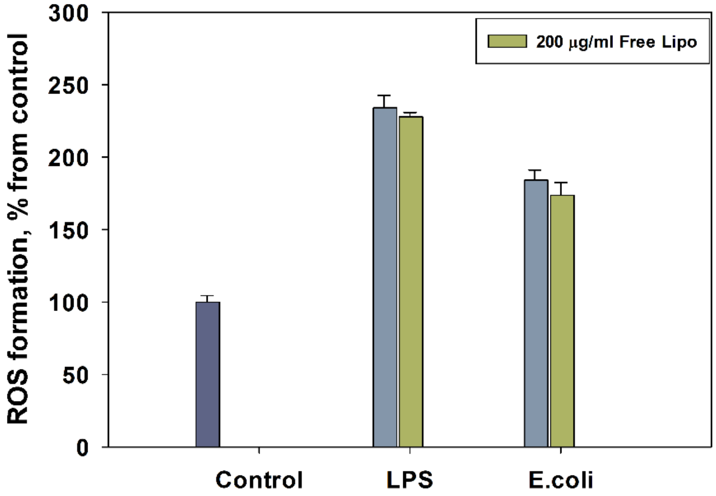

Figure S2. The effects of free liposomes (200 μg/ml) on the levels of ROS. Cells (1 × 10^4 cells/well) were incubated with LPS at 1.0 μg/mL or *E. coli* 1 × 10^2 CFU for 24 h.

Table S1. Summary of the anti-inflammatory activity of liposomes containing CRG/ECh complex or CRG when keratinocytes were treated with LPS or *E. coli* for 24 h.

	Control	LPS	*E.coli*	GRG/Ech 200	GRG/Ech 100	GRG/Ech 50	CRG 200	CRG 100	CRG 50
Lysosomal activity	100.0±2.9	-	122.6±4.2	103.4±16.4	103.4±13.2	119.1±16.4	111.6±5.1	118.4±10.2	113.6±10.9
	100.0±3.3	184.6±8.6	-	132.1±4.2	157.8±0.9	150.5±8.2	134.7±3.7	138.9±9.1	152.6±3.3
ROS	100.0±4.4	-	184.2±7.0	147.3±1.7	136.6±12.9	142.3±6.5	152.6±3.8	141.8±3.5	154.3±4.1
	100.0±4.4	234.1±8.6		205.3±9.0	181.9±14.7	197.9±2.1	195.1±11.3	192.5±1.5	197.7±6.7
NO	100.0±1.5		116.0±1.8	121.0±1.3	116.2±6.8	111.1±8.3	107.5±0.5	106.8±8.9	119.3±6.8
	100.0±1.5	118.4±4.2		116.5±5.1	113.4±8.8	111.0±8.6	117.2±0.7	115.6±4.5	112.9±4.7
Migration	98.9±0.1	34.2±1.8	-	65.6±2.5	70.1±1.4	-	-	-	-
